# 2-Ferrocenyl-3-meth­oxy-6-methyl­pyridine

**DOI:** 10.1107/S1600536809012288

**Published:** 2009-04-10

**Authors:** Chen Xu, Xin-Qi Hao, Fang Liu, Xiu-Juan Wu, Mao-Ping Song

**Affiliations:** a College of Chemistry and Chemical Engineering, Luoyang Normal University, Luoyang 471022, People’s Republic of China; b Department of Chemistry, Henan Key Laboratory of Chemical, Biology and Organic Chemistry, Zhengzhou University, Zhengzhou 450052, People’s Republic of China

## Abstract

In the title compound, [Fe(C_5_H_5_)(C_12_H_12_NO)], the dihedral angle between the pyridyl and substituted cyclo­penta­dienyl rings is 23.58 (3)°. The crystal structure is characterized by weak inter­molecular C—H⋯N hydrogen-bonding contacts, leading to the formation of chains running parallel to the *n*-glide planes. A weak inter­molecular C—H⋯π contact is also present.

## Related literature

For historical background and for properties of ferrocenes and derivatives, see: Wang *et al.* (2008 ▶) and references cited therein. For the structure of (*Z*)-2,3-di(ferrocen­yl)-2-butenedionate, see: Beletskaya *et al.* (2001 ▶). For cyclo­palladated ferrocen­yl–pyrimidine complexes, see: Xu *et al.* (2009 ▶). For the structure of {1-[(3,5-dimethyl-4*H*-1,2,4-triazol-4-yl)-imino]eth­yl}ferrocene, see: Hao *et al.* (2008 ▶). For the synthesis of functional compounds related to ferrocene-bearing units, see: Sarhan & Izumi (2003 ▶).
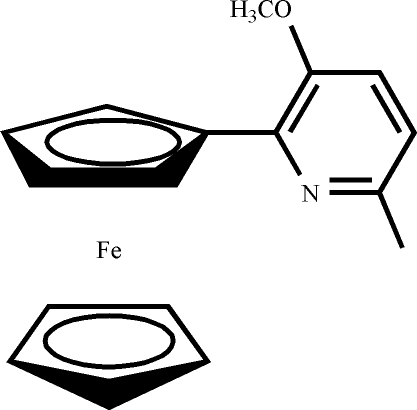


## Experimental

### 

#### Crystal data

[Fe(C_5_H_5_)(C_12_H_12_NO)]
*M* *_r_* = 307.17
Monoclinic, 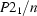

*a* = 5.9949 (13) Å
*b* = 20.284 (4) Å
*c* = 12.035 (2) Å
β = 100.036 (3)°
*V* = 1441.0 (5) Å^3^
*Z* = 4
Mo *K*α radiation
μ = 1.04 mm^−1^
*T* = 294 K
0.43 × 0.35 × 0.27 mm

#### Data collection

Bruker SMART APEX CCD area-detector diffractometer
Absorption correction: multi-scan (*SADABS*; Sheldrick, 1996 ▶) *T*_min_ = 0.663, *T*_max_ = 0.767
8219 measured reflections
2673 independent reflections
2280 reflections with *I* > 2σ(*I*)
*R*_int_ = 0.020

#### Refinement

*R*[*F* ^2^ > 2σ(*F* ^2^)] = 0.029
*wR*(*F* ^2^) = 0.076
*S* = 1.06
2673 reflections
183 parameters
H-atom parameters constrained
Δρ_max_ = 0.23 e Å^−3^
Δρ_min_ = −0.27 e Å^−3^

### 

Data collection: *SMART* (Bruker, 2004 ▶); cell refinement: *SAINT* (Bruker, 2004 ▶); data reduction: *SAINT*; program(s) used to solve structure: *SHELXS97* (Sheldrick, 2008 ▶); program(s) used to refine structure: *SHELXL97* (Sheldrick, 2008 ▶); molecular graphics: *SHELXTL* (Sheldrick, 2008 ▶); software used to prepare material for publication: *SHELXTL* .

## Supplementary Material

Crystal structure: contains datablocks global, I. DOI: 10.1107/S1600536809012288/si2164sup1.cif

Structure factors: contains datablocks I. DOI: 10.1107/S1600536809012288/si2164Isup2.hkl

Additional supplementary materials:  crystallographic information; 3D view; checkCIF report

## Figures and Tables

**Table 1:** Hydrogen-bond geometry (Å, °)

*D*—H⋯*A*	*D*—H	H⋯*A*	*D*⋯*A*	*D*—H⋯*A*
C3—H3⋯N1^i^	0.93	2.65	3.577 (3)	172
C4—H4⋯*Cg*1^ii^	0.93	2.96	3.880 (3)	173

Symmetry codes: (i) 
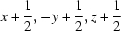
; (ii) 
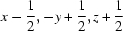
. *Cg*1 is the centroid of the C8–C12 cyclo­penta­dienyl ring.

**Fig. 1.**
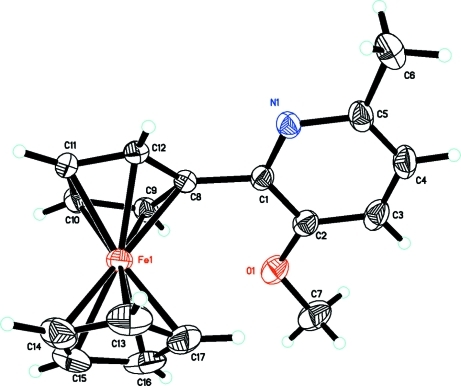
The molecular structure of the title compound with displacement ellipsoids drawn at the 30% probability level.

**Fig. 2.**
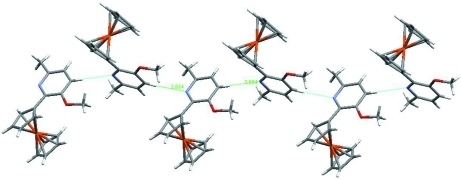
Partial view of the crystal packing showing the formation of the one-dimensional chain structure formed by the weak intermolecular C—H···N hydrogen bonding contacts.

## supplementary crystallographic information

### Comment

Since the discovery of ferrocene in the 1950s, the fascinating structural properties of ferrocene and its derivatives have been the subject of increasing interest in all fields of organometallic chemistry (Hao *et al.*, 2008; Xu *et al.*, 2009; Wang *et al.*, 2008 with relevant literature cited therein). Among them, ferrocene-heterocycles are one of the most important ones (Sarhan & Izumi, 2003).

In the title compound (Fig. 1), the dihedral angle between the pyridyl and substituted cyclopentadienyl rings is 23.58 (3)°. The crystal structure is characterised by weak intermolecular C—H···N hydrogen bonding contacts (Table 1), leading to the formation of one-dimensional chains running parallel to the n-glide planes (Fig. 2). Furthermore, a weak intermolecular C—H···π contact may also be considered in the structure (Table 1). Cg1 is the centroid of the Cp ring C8 - C12. The perpendicular distance of H4 to the Cp ring is 2.812 Å. C—H···π contacts were also observed in a triazol-ferrocene derivative (Hao *et al.*, 2008). The n-glide plane symmetry operation is also observed in the structure of 2-Ferrocenyl-6-methylpyridin-3-ol (Wang *et al.*, 2008), in which the nitrogen atoms form classic intermolecular O—H···N hydrogen bonds with the adjacent -OH groups. Both compounds crystallize in the space group P2_1_/n .

### Experimental

The title compound was prepared as described in the literature (Beletskaya *et al.*, 2001; Xu *et al.*, 2009) and recrystallized from dichloromethane-petroleum ether solution at room temperature to give the desired product as red crystals.

### Refinement

H atoms attached to C atoms of the title compound were placed in geometrically idealized positions and treated as riding with C—H distances constrained to 0.93–0.96 Å, and with *U*_iso_(H)=1.2*U*_eq_(C) (1.5*U*_eq_ for methyl H).

### Crystal data

**Table:**

[Fe(C_5_H_5_)(C_12_H_12_NO)]	*F*(000) = 640
*M**_r_* = 307.17	*D*_x_ = 1.416 Mg m^−^^3^
Monoclinic, *P*2_1_/*n*	Mo *K*α radiation, λ = 0.71073 Å
Hall symbol: -P 2yn	Cell parameters from 3338 reflections
*a* = 5.9949 (13) Å	θ = 2.7–26.2°
*b* = 20.284 (4) Å	µ = 1.04 mm^−^^1^
*c* = 12.035 (2) Å	*T* = 294 K
β = 100.036 (3)°	Block, red
*V* = 1441.0 (5) Å^3^	0.43 × 0.35 × 0.27 mm
*Z* = 4	

### Data collection

**Table:**

Bruker SMART APEX CCD area-detector diffractometer	2673 independent reflections
Radiation source: fine-focus sealed tube	2280 reflections with *I* > 2σ(*I*)
graphite	*R*_int_ = 0.020
φ and ω scans	θ_max_ = 25.5°, θ_min_ = 2.6°
Absorption correction: multi-scan (*SADABS*; Sheldrick, 1996)	*h* = −7→7
*T*_min_ = 0.663, *T*_max_ = 0.767	*k* = −24→23
8219 measured reflections	*l* = −14→10

### Refinement

**Table:**

Refinement on *F*^2^	Primary atom site location: structure-invariant direct methods
Least-squares matrix: full	Secondary atom site location: difference Fourier map
*R*[*F*^2^ > 2σ(*F*^2^)] = 0.029	Hydrogen site location: inferred from neighbouring sites
*wR*(*F*^2^) = 0.076	H-atom parameters constrained
*S* = 1.06	*w* = 1/[σ^2^(*F*_o_^2^) + (0.0389*P*)^2^ + 0.3178*P*] where *P* = (*F*_o_^2^ + 2*F*_c_^2^)/3
2673 reflections	(Δ/σ)_max_ < 0.001
183 parameters	Δρ_max_ = 0.23 e Å^−^^3^
0 restraints	Δρ_min_ = −0.27 e Å^−^^3^

### Special details

**Table:**

Geometry. All e.s.d.'s (except the e.s.d. in the dihedral angle between two l.s. planes)are estimated using the full covariance matrix. The cell e.s.d.'s are takeninto account individually in the estimation of e.s.d.'s in distances, anglesand torsion angles; correlations between e.s.d.'s in cell parameters are onlyused when they are defined by crystal symmetry. An approximate (isotropic)treatment of cell e.s.d.'s is used for estimating e.s.d.'s involving l.s. planes.
Refinement. Refinement of *F*^2^ against ALL reflections. The weighted *R*-factor *wR* andgoodness of fit *S* are based on *F*^2^, conventional *R*-factors *R* are basedon *F*, with *F* set to zero for negative *F*^2^. The threshold expression of*F*^2^ > σ(*F*^2^) is used only for calculating *R*-factors(gt) *etc*. and isnot relevant to the choice of reflections for refinement. *R*-factors basedon *F*^2^ are statistically about twice as large as those based on *F*, and *R*-factors based on ALL data will be even larger.

### Fractional atomic coordinates and isotropic or equivalent isotropic displacement parameters (Å^2^)

**Table:**

	*x*	*y*	*z*	*U*_iso_*/*U*_eq_	
C1	0.2209 (3)	0.19049 (9)	0.27110 (15)	0.0381 (4)	
C2	0.3488 (4)	0.20189 (10)	0.37922 (17)	0.0454 (5)	
C3	0.2693 (4)	0.24755 (11)	0.44894 (18)	0.0555 (6)	
H3	0.3500	0.2563	0.5207	0.067*	
C4	0.0683 (4)	0.27974 (10)	0.4098 (2)	0.0576 (6)	
H4	0.0137	0.3108	0.4552	0.069*	
C5	−0.0523 (4)	0.26628 (9)	0.30382 (18)	0.0483 (5)	
C6	−0.2728 (4)	0.29957 (11)	0.2575 (2)	0.0636 (6)	
H6A	−0.2499	0.3305	0.2002	0.095*	
H6B	−0.3277	0.3224	0.3172	0.095*	
H6C	−0.3818	0.2671	0.2255	0.095*	
C7	0.6707 (4)	0.17331 (14)	0.5189 (2)	0.0729 (7)	
H7A	0.7117	0.2187	0.5333	0.109*	
H7B	0.8054	0.1469	0.5263	0.109*	
H7C	0.5794	0.1587	0.5722	0.109*	
C8	0.2889 (3)	0.14341 (9)	0.18926 (15)	0.0375 (4)	
C9	0.5096 (3)	0.11890 (9)	0.18035 (17)	0.0416 (4)	
H9	0.6521	0.1303	0.2298	0.050*	
C10	0.4844 (4)	0.07527 (9)	0.08687 (17)	0.0461 (5)	
H10	0.6071	0.0510	0.0611	0.055*	
C11	0.2523 (4)	0.07186 (10)	0.03813 (17)	0.0477 (5)	
H11	0.1868	0.0450	−0.0271	0.057*	
C12	0.1309 (3)	0.11391 (9)	0.10025 (16)	0.0415 (4)	
H12	−0.0333	0.1210	0.0856	0.050*	
C13	0.0699 (4)	−0.01181 (13)	0.2693 (2)	0.0722 (8)	
H13	−0.0952	−0.0068	0.2529	0.087*	
C14	0.1998 (5)	−0.05346 (11)	0.2134 (2)	0.0665 (7)	
H14	0.1411	−0.0819	0.1491	0.080*	
C15	0.4275 (5)	−0.04656 (11)	0.2624 (3)	0.0683 (7)	
H15	0.5550	−0.0696	0.2386	0.082*	
C16	0.4429 (5)	−0.00088 (13)	0.3495 (2)	0.0722 (8)	
H16	0.5830	0.0132	0.3984	0.087*	
C17	0.2229 (6)	0.02140 (14)	0.3562 (2)	0.0769 (8)	
H17	0.1820	0.0530	0.4109	0.092*	
Fe1	0.29948 (4)	0.042837 (12)	0.20323 (2)	0.03896 (11)	
N1	0.0252 (3)	0.22223 (7)	0.23573 (14)	0.0416 (4)	
O1	0.5451 (3)	0.16693 (8)	0.40698 (12)	0.0597 (4)	

### Atomic displacement parameters (Å^2^)

**Table:**

	*U*^11^	*U*^22^	*U*^33^	*U*^12^	*U*^13^	*U*^23^
C1	0.0488 (11)	0.0324 (9)	0.0342 (10)	−0.0068 (8)	0.0100 (9)	−0.0025 (8)
C2	0.0538 (12)	0.0421 (10)	0.0399 (11)	−0.0080 (9)	0.0073 (9)	−0.0020 (9)
C3	0.0814 (16)	0.0475 (12)	0.0389 (12)	−0.0181 (11)	0.0144 (11)	−0.0122 (10)
C4	0.0811 (17)	0.0412 (11)	0.0560 (14)	−0.0047 (11)	0.0269 (13)	−0.0144 (10)
C5	0.0627 (13)	0.0330 (10)	0.0549 (13)	−0.0036 (9)	0.0255 (11)	−0.0037 (9)
C6	0.0650 (15)	0.0465 (13)	0.0848 (18)	0.0062 (11)	0.0283 (13)	−0.0044 (12)
C7	0.0755 (17)	0.0905 (19)	0.0452 (14)	−0.0078 (14)	−0.0108 (12)	−0.0013 (13)
C8	0.0477 (10)	0.0313 (9)	0.0336 (10)	0.0011 (7)	0.0073 (8)	0.0007 (7)
C9	0.0451 (10)	0.0355 (10)	0.0449 (12)	−0.0024 (8)	0.0096 (9)	0.0001 (8)
C10	0.0565 (12)	0.0390 (11)	0.0456 (12)	0.0068 (9)	0.0166 (10)	−0.0004 (9)
C11	0.0680 (14)	0.0393 (10)	0.0330 (11)	0.0080 (9)	0.0014 (10)	−0.0046 (8)
C12	0.0491 (11)	0.0379 (10)	0.0345 (10)	0.0061 (8)	−0.0011 (8)	0.0011 (8)
C13	0.0561 (14)	0.0766 (17)	0.085 (2)	−0.0117 (13)	0.0144 (14)	0.0298 (15)
C14	0.0759 (17)	0.0432 (12)	0.0765 (19)	−0.0133 (11)	0.0025 (14)	0.0082 (12)
C15	0.0702 (16)	0.0464 (13)	0.084 (2)	0.0045 (11)	0.0009 (14)	0.0224 (13)
C16	0.0786 (18)	0.0742 (17)	0.0542 (16)	−0.0149 (14)	−0.0153 (13)	0.0319 (14)
C17	0.127 (3)	0.0601 (15)	0.0502 (15)	−0.0075 (16)	0.0338 (16)	0.0152 (12)
Fe1	0.04316 (17)	0.03381 (16)	0.03740 (18)	−0.00120 (11)	0.00003 (12)	0.00266 (11)
N1	0.0508 (9)	0.0325 (8)	0.0433 (9)	−0.0001 (7)	0.0133 (8)	−0.0012 (7)
O1	0.0649 (10)	0.0689 (10)	0.0398 (9)	0.0010 (8)	−0.0063 (7)	−0.0086 (7)

### Geometric parameters (Å, °)

**Table:**

C1—N1	1.341 (2)	C10—C11	1.415 (3)
C1—C2	1.410 (3)	C10—Fe1	2.041 (2)
C1—C8	1.479 (2)	C10—H10	0.9800
C2—O1	1.364 (3)	C11—C12	1.416 (3)
C2—C3	1.389 (3)	C11—Fe1	2.044 (2)
C3—C4	1.379 (3)	C11—H11	0.9800
C3—H3	0.9300	C12—Fe1	2.0492 (18)
C4—C5	1.379 (3)	C12—H12	0.9800
C4—H4	0.9300	C13—C14	1.399 (4)
C5—N1	1.349 (2)	C13—C17	1.434 (4)
C5—C6	1.503 (3)	C13—Fe1	2.034 (2)
C6—H6A	0.9600	C13—H13	0.9800
C6—H6B	0.9600	C14—C15	1.396 (4)
C6—H6C	0.9600	C14—Fe1	2.053 (2)
C7—O1	1.430 (3)	C14—H14	0.9800
C7—H7A	0.9600	C15—C16	1.390 (4)
C7—H7B	0.9600	C15—Fe1	2.048 (2)
C7—H7C	0.9600	C15—H15	0.9800
C8—C12	1.431 (3)	C16—C17	1.410 (4)
C8—C9	1.435 (3)	C16—Fe1	2.024 (2)
C8—Fe1	2.0470 (18)	C16—H16	0.9800
C9—C10	1.419 (3)	C17—Fe1	2.021 (2)
C9—Fe1	2.0405 (18)	C17—H17	0.9800
C9—H9	0.9800		
			
N1—C1—C2	121.29 (17)	C13—C14—H14	125.6
N1—C1—C8	115.20 (16)	Fe1—C14—H14	125.6
C2—C1—C8	123.50 (18)	C16—C15—C14	108.5 (3)
O1—C2—C3	124.81 (19)	C16—C15—Fe1	69.12 (13)
O1—C2—C1	116.61 (17)	C14—C15—Fe1	70.28 (13)
C3—C2—C1	118.6 (2)	C16—C15—H15	125.8
C4—C3—C2	118.7 (2)	C14—C15—H15	125.8
C4—C3—H3	120.6	Fe1—C15—H15	125.8
C2—C3—H3	120.6	C15—C16—C17	108.5 (2)
C3—C4—C5	120.5 (2)	C15—C16—Fe1	70.96 (14)
C3—C4—H4	119.7	C17—C16—Fe1	69.50 (13)
C5—C4—H4	119.7	C15—C16—H16	125.8
N1—C5—C4	120.8 (2)	C17—C16—H16	125.8
N1—C5—C6	116.5 (2)	Fe1—C16—H16	125.8
C4—C5—C6	122.67 (19)	C16—C17—C13	107.0 (3)
C5—C6—H6A	109.5	C16—C17—Fe1	69.70 (15)
C5—C6—H6B	109.5	C13—C17—Fe1	69.76 (14)
H6A—C6—H6B	109.5	C16—C17—H17	126.5
C5—C6—H6C	109.5	C13—C17—H17	126.5
H6A—C6—H6C	109.5	Fe1—C17—H17	126.5
H6B—C6—H6C	109.5	C17—Fe1—C16	40.81 (12)
O1—C7—H7A	109.5	C17—Fe1—C13	41.42 (11)
O1—C7—H7B	109.5	C16—Fe1—C13	68.62 (11)
H7A—C7—H7B	109.5	C17—Fe1—C10	158.66 (12)
O1—C7—H7C	109.5	C16—Fe1—C10	122.60 (10)
H7A—C7—H7C	109.5	C13—Fe1—C10	158.36 (11)
H7B—C7—H7C	109.5	C17—Fe1—C9	121.95 (11)
C12—C8—C9	107.31 (16)	C16—Fe1—C9	105.66 (9)
C12—C8—C1	123.04 (17)	C13—Fe1—C9	160.15 (11)
C9—C8—C1	129.64 (17)	C10—Fe1—C9	40.68 (8)
C12—C8—Fe1	69.63 (10)	C17—Fe1—C11	158.83 (12)
C9—C8—Fe1	69.20 (10)	C16—Fe1—C11	159.61 (11)
C1—C8—Fe1	126.65 (13)	C13—Fe1—C11	123.25 (10)
C10—C9—C8	107.66 (17)	C10—Fe1—C11	40.52 (8)
C10—C9—Fe1	69.66 (11)	C9—Fe1—C11	68.61 (8)
C8—C9—Fe1	69.69 (10)	C17—Fe1—C8	106.28 (10)
C10—C9—H9	126.2	C16—Fe1—C8	120.70 (10)
C8—C9—H9	126.2	C13—Fe1—C8	124.18 (10)
Fe1—C9—H9	126.2	C10—Fe1—C8	68.61 (7)
C11—C10—C9	108.69 (17)	C9—Fe1—C8	41.11 (7)
C11—C10—Fe1	69.88 (12)	C11—Fe1—C8	68.66 (7)
C9—C10—Fe1	69.66 (11)	C17—Fe1—C15	67.90 (12)
C11—C10—H10	125.7	C16—Fe1—C15	39.92 (11)
C9—C10—H10	125.7	C13—Fe1—C15	67.65 (11)
Fe1—C10—H10	125.7	C10—Fe1—C15	107.97 (10)
C10—C11—C12	108.09 (17)	C9—Fe1—C15	120.91 (9)
C10—C11—Fe1	69.60 (11)	C11—Fe1—C15	124.95 (10)
C12—C11—Fe1	69.95 (11)	C8—Fe1—C15	156.28 (9)
C10—C11—H11	126.0	C17—Fe1—C12	122.41 (11)
C12—C11—H11	126.0	C16—Fe1—C12	157.56 (11)
Fe1—C11—H11	126.0	C13—Fe1—C12	108.88 (9)
C11—C12—C8	108.25 (17)	C10—Fe1—C12	68.15 (8)
C11—C12—Fe1	69.57 (11)	C9—Fe1—C12	68.74 (8)
C8—C12—Fe1	69.46 (10)	C11—Fe1—C12	40.48 (8)
C11—C12—H12	125.9	C8—Fe1—C12	40.91 (7)
C8—C12—H12	125.9	C15—Fe1—C12	161.55 (10)
Fe1—C12—H12	125.9	C17—Fe1—C14	68.07 (12)
C14—C13—C17	107.2 (2)	C16—Fe1—C14	67.38 (10)
C14—C13—Fe1	70.71 (14)	C13—Fe1—C14	40.03 (11)
C17—C13—Fe1	68.82 (14)	C10—Fe1—C14	123.10 (10)
C14—C13—H13	126.4	C9—Fe1—C14	157.00 (10)
C17—C13—H13	126.4	C11—Fe1—C14	109.84 (10)
Fe1—C13—H13	126.4	C8—Fe1—C14	161.46 (10)
C15—C14—C13	108.8 (2)	C15—Fe1—C14	39.82 (10)
C15—C14—Fe1	69.90 (13)	C12—Fe1—C14	125.97 (9)
C13—C14—Fe1	69.27 (13)	C1—N1—C5	120.02 (17)
C15—C14—H14	125.6	C2—O1—C7	118.28 (18)
			
N1—C1—C2—O1	179.85 (17)	C9—C10—Fe1—C14	−158.02 (13)
C8—C1—C2—O1	0.2 (3)	C10—C9—Fe1—C17	163.42 (14)
N1—C1—C2—C3	−0.7 (3)	C8—C9—Fe1—C17	−77.75 (16)
C8—C1—C2—C3	179.65 (18)	C10—C9—Fe1—C16	122.15 (15)
O1—C2—C3—C4	179.62 (19)	C8—C9—Fe1—C16	−119.02 (14)
C1—C2—C3—C4	0.2 (3)	C10—C9—Fe1—C13	−167.6 (3)
C2—C3—C4—C5	0.6 (3)	C8—C9—Fe1—C13	−48.8 (3)
C3—C4—C5—N1	−1.0 (3)	C8—C9—Fe1—C10	118.83 (16)
C3—C4—C5—C6	179.5 (2)	C10—C9—Fe1—C11	−37.19 (12)
N1—C1—C8—C12	−23.2 (3)	C8—C9—Fe1—C11	81.64 (12)
C2—C1—C8—C12	156.51 (18)	C10—C9—Fe1—C8	−118.83 (16)
N1—C1—C8—C9	156.33 (18)	C10—C9—Fe1—C15	81.66 (16)
C2—C1—C8—C9	−24.0 (3)	C8—C9—Fe1—C15	−159.51 (13)
N1—C1—C8—Fe1	−111.27 (17)	C10—C9—Fe1—C12	−80.79 (13)
C2—C1—C8—Fe1	68.4 (2)	C8—C9—Fe1—C12	38.04 (11)
C12—C8—C9—C10	0.1 (2)	C10—C9—Fe1—C14	53.4 (3)
C1—C8—C9—C10	−179.48 (18)	C8—C9—Fe1—C14	172.2 (2)
Fe1—C8—C9—C10	59.55 (13)	C10—C11—Fe1—C17	161.5 (3)
C12—C8—C9—Fe1	−59.44 (13)	C12—C11—Fe1—C17	42.3 (3)
C1—C8—C9—Fe1	121.0 (2)	C10—C11—Fe1—C16	−39.8 (3)
C8—C9—C10—C11	−0.4 (2)	C12—C11—Fe1—C16	−159.1 (2)
Fe1—C9—C10—C11	59.16 (14)	C10—C11—Fe1—C13	−160.67 (14)
C8—C9—C10—Fe1	−59.57 (13)	C12—C11—Fe1—C13	80.10 (16)
C9—C10—C11—C12	0.6 (2)	C12—C11—Fe1—C10	−119.24 (17)
Fe1—C10—C11—C12	59.58 (14)	C10—C11—Fe1—C9	37.33 (11)
C9—C10—C11—Fe1	−59.02 (14)	C12—C11—Fe1—C9	−81.91 (12)
C10—C11—C12—C8	−0.5 (2)	C10—C11—Fe1—C8	81.62 (12)
Fe1—C11—C12—C8	58.87 (13)	C12—C11—Fe1—C8	−37.61 (11)
C10—C11—C12—Fe1	−59.36 (14)	C10—C11—Fe1—C15	−76.21 (15)
C9—C8—C12—C11	0.2 (2)	C12—C11—Fe1—C15	164.55 (13)
C1—C8—C12—C11	179.86 (17)	C10—C11—Fe1—C12	119.24 (17)
Fe1—C8—C12—C11	−58.94 (13)	C10—C11—Fe1—C14	−118.13 (13)
C9—C8—C12—Fe1	59.17 (13)	C12—C11—Fe1—C14	122.63 (14)
C1—C8—C12—Fe1	−121.20 (17)	C12—C8—Fe1—C17	−121.03 (15)
C17—C13—C14—C15	−0.5 (3)	C9—C8—Fe1—C17	120.25 (15)
Fe1—C13—C14—C15	58.89 (17)	C1—C8—Fe1—C17	−4.4 (2)
C17—C13—C14—Fe1	−59.40 (16)	C12—C8—Fe1—C16	−162.99 (14)
C13—C14—C15—C16	0.2 (3)	C9—C8—Fe1—C16	78.29 (15)
Fe1—C14—C15—C16	58.73 (16)	C1—C8—Fe1—C16	−46.3 (2)
C13—C14—C15—Fe1	−58.50 (17)	C12—C8—Fe1—C13	−79.27 (15)
C14—C15—C16—C17	0.2 (3)	C9—C8—Fe1—C13	162.01 (13)
Fe1—C15—C16—C17	59.60 (16)	C1—C8—Fe1—C13	37.4 (2)
C14—C15—C16—Fe1	−59.44 (17)	C12—C8—Fe1—C10	80.89 (13)
C15—C16—C17—C13	−0.5 (3)	C9—C8—Fe1—C10	−37.83 (12)
Fe1—C16—C17—C13	60.05 (17)	C1—C8—Fe1—C10	−162.45 (18)
C15—C16—C17—Fe1	−60.51 (17)	C12—C8—Fe1—C9	118.72 (16)
C14—C13—C17—C16	0.6 (3)	C1—C8—Fe1—C9	−124.6 (2)
Fe1—C13—C17—C16	−60.01 (16)	C12—C8—Fe1—C11	37.23 (12)
C14—C13—C17—Fe1	60.60 (17)	C9—C8—Fe1—C11	−81.49 (12)
C13—C17—Fe1—C16	−118.0 (2)	C1—C8—Fe1—C11	153.89 (19)
C16—C17—Fe1—C13	118.0 (2)	C12—C8—Fe1—C15	167.0 (2)
C16—C17—Fe1—C10	−45.6 (3)	C9—C8—Fe1—C15	48.3 (3)
C13—C17—Fe1—C10	−163.6 (2)	C1—C8—Fe1—C15	−76.3 (3)
C16—C17—Fe1—C9	−76.37 (18)	C9—C8—Fe1—C12	−118.72 (16)
C13—C17—Fe1—C9	165.62 (15)	C1—C8—Fe1—C12	116.7 (2)
C16—C17—Fe1—C11	168.8 (2)	C12—C8—Fe1—C14	−51.7 (3)
C13—C17—Fe1—C11	50.8 (3)	C9—C8—Fe1—C14	−170.4 (3)
C16—C17—Fe1—C8	−118.39 (16)	C1—C8—Fe1—C14	65.0 (4)
C13—C17—Fe1—C8	123.61 (16)	C16—C15—Fe1—C17	−38.02 (17)
C16—C17—Fe1—C15	37.22 (16)	C14—C15—Fe1—C17	81.80 (19)
C13—C17—Fe1—C15	−80.78 (17)	C14—C15—Fe1—C16	119.8 (3)
C16—C17—Fe1—C12	−160.05 (15)	C16—C15—Fe1—C13	−82.94 (19)
C13—C17—Fe1—C12	81.95 (18)	C14—C15—Fe1—C13	36.89 (18)
C16—C17—Fe1—C14	80.32 (18)	C16—C15—Fe1—C10	119.66 (17)
C13—C17—Fe1—C14	−37.68 (16)	C14—C15—Fe1—C10	−120.51 (17)
C15—C16—Fe1—C17	119.2 (2)	C16—C15—Fe1—C9	76.98 (19)
C15—C16—Fe1—C13	80.30 (18)	C14—C15—Fe1—C9	−163.20 (16)
C17—C16—Fe1—C13	−38.85 (17)	C16—C15—Fe1—C11	161.22 (16)
C15—C16—Fe1—C10	−78.84 (18)	C14—C15—Fe1—C11	−78.9 (2)
C17—C16—Fe1—C10	162.01 (15)	C16—C15—Fe1—C8	42.1 (3)
C15—C16—Fe1—C9	−119.76 (16)	C14—C15—Fe1—C8	161.9 (2)
C17—C16—Fe1—C9	121.09 (16)	C16—C15—Fe1—C12	−165.7 (3)
C15—C16—Fe1—C11	−49.2 (3)	C14—C15—Fe1—C12	−45.8 (4)
C17—C16—Fe1—C11	−168.4 (2)	C16—C15—Fe1—C14	−119.8 (3)
C15—C16—Fe1—C8	−161.72 (15)	C11—C12—Fe1—C17	−163.27 (15)
C17—C16—Fe1—C8	79.13 (18)	C8—C12—Fe1—C17	76.97 (16)
C17—C16—Fe1—C15	−119.2 (2)	C11—C12—Fe1—C16	161.0 (2)
C15—C16—Fe1—C12	168.2 (2)	C8—C12—Fe1—C16	41.2 (3)
C17—C16—Fe1—C12	49.0 (3)	C11—C12—Fe1—C13	−119.46 (15)
C15—C16—Fe1—C14	37.00 (17)	C8—C12—Fe1—C13	120.79 (14)
C17—C16—Fe1—C14	−82.15 (18)	C11—C12—Fe1—C10	37.65 (12)
C14—C13—Fe1—C17	−118.2 (2)	C8—C12—Fe1—C10	−82.10 (12)
C14—C13—Fe1—C16	−79.86 (17)	C11—C12—Fe1—C9	81.54 (13)
C17—C13—Fe1—C16	38.29 (17)	C8—C12—Fe1—C9	−38.22 (11)
C14—C13—Fe1—C10	45.7 (3)	C8—C12—Fe1—C11	−119.76 (17)
C17—C13—Fe1—C10	163.9 (2)	C11—C12—Fe1—C8	119.76 (17)
C14—C13—Fe1—C9	−156.5 (2)	C11—C12—Fe1—C15	−43.6 (4)
C17—C13—Fe1—C9	−38.4 (3)	C8—C12—Fe1—C15	−163.4 (3)
C14—C13—Fe1—C11	81.39 (17)	C11—C12—Fe1—C14	−78.20 (17)
C17—C13—Fe1—C11	−160.45 (16)	C8—C12—Fe1—C14	162.04 (13)
C14—C13—Fe1—C8	166.76 (14)	C15—C14—Fe1—C17	−81.35 (19)
C17—C13—Fe1—C8	−75.09 (18)	C13—C14—Fe1—C17	38.96 (17)
C14—C13—Fe1—C15	−36.71 (16)	C15—C14—Fe1—C16	−37.10 (18)
C17—C13—Fe1—C15	81.45 (18)	C13—C14—Fe1—C16	83.22 (18)
C14—C13—Fe1—C12	123.92 (15)	C15—C14—Fe1—C13	−120.3 (2)
C17—C13—Fe1—C12	−117.93 (17)	C15—C14—Fe1—C10	78.06 (19)
C17—C13—Fe1—C14	118.2 (2)	C13—C14—Fe1—C10	−161.63 (15)
C11—C10—Fe1—C17	−161.7 (3)	C15—C14—Fe1—C9	39.4 (3)
C9—C10—Fe1—C17	−41.7 (3)	C13—C14—Fe1—C9	159.7 (2)
C11—C10—Fe1—C16	164.63 (13)	C15—C14—Fe1—C11	121.21 (17)
C9—C10—Fe1—C16	−75.38 (16)	C13—C14—Fe1—C11	−118.47 (16)
C11—C10—Fe1—C13	48.7 (3)	C15—C14—Fe1—C8	−156.9 (3)
C9—C10—Fe1—C13	168.6 (2)	C13—C14—Fe1—C8	−36.6 (4)
C11—C10—Fe1—C9	−119.99 (16)	C13—C14—Fe1—C15	120.3 (2)
C9—C10—Fe1—C11	119.99 (16)	C15—C14—Fe1—C12	163.71 (16)
C11—C10—Fe1—C8	−81.77 (12)	C13—C14—Fe1—C12	−75.98 (18)
C9—C10—Fe1—C8	38.22 (11)	C2—C1—N1—C5	0.3 (3)
C11—C10—Fe1—C15	123.19 (13)	C8—C1—N1—C5	−179.98 (16)
C9—C10—Fe1—C15	−116.83 (13)	C4—C5—N1—C1	0.5 (3)
C11—C10—Fe1—C12	−37.61 (11)	C6—C5—N1—C1	180.00 (17)
C9—C10—Fe1—C12	82.37 (12)	C3—C2—O1—C7	5.3 (3)
C11—C10—Fe1—C14	81.99 (15)	C1—C2—O1—C7	−175.28 (19)

### Hydrogen-bond geometry (Å, °)

**Table:**

*D*—H···*A*	*D*—H	H···*A*	*D*···*A*	*D*—H···*A*
C3—H3···N1^i^	0.93	2.65	3.577 (3)	172
C4—H4···Cg1^ii^	0.93	2.96	3.880 (3)	173

Symmetry codes: (i) *x*+1/2, −*y*+1/2, *z*+1/2; (ii) *x*−1/2, −*y*+1/2, *z*+1/2.
